# Correlation of Acceleration Curves in Gravitational Direction for Different Body Segments during High-Impact Jumping Exercises

**DOI:** 10.3390/s23042276

**Published:** 2023-02-17

**Authors:** Lukas Reinker, Dominic Bläsing, Rudolf Bierl, Sabina Ulbricht, Sebastian Dendorfer

**Affiliations:** 1 Laboratory for Biomechanics, OTH Regensburg, 93053 Regensburg, Germany; 2 Regensburg Center of Biomedical Engineering (RCBE), OTH Regensburg and University of Regensburg, 93053 Regensburg, Germany; 3 Department of Prevention Research and Social Medicine, Institute for Community Medicine, University Medicine Greifswald, Walther-Rathenau-Str. 48, 17475 Greifswald, Germany; 4 German Centre for Cardiovascular Research (DZHK), Partner Site Greifswald, 17475 Greifswald, Germany; 5 Sensorik-ApplikationsZentrum, OTH Regensburg, 93053 Regensburg, Germany

**Keywords:** osteoporosis, inertial measurement units, wearable motion-tracking sensors, high-impact exercises, jumping exercises

## Abstract

Osteoporosis is a common disease of old age. However, in many cases, it can be very well prevented and counteracted with physical activity, especially high-impact exercises. Wearables have the potential to provide data that can help with continuous monitoring of patients during therapy phases or preventive exercise programs in everyday life. This study aimed to determine the accuracy and reliability of measured acceleration data at different body positions compared to accelerations at the pelvis during different jumping exercises. Accelerations at the hips have been investigated in previous studies with regard to osteoporosis prevention. Data were collected using an IMU-based motion capture system (Xsens) consisting of 17 sensors. Forty-nine subjects were included in this study. The analysis shows the correlation between impacts and the corresponding drop height, which are dependent on the respective exercise. Very high correlations (0.83–0.94) were found between accelerations at the pelvis and the other measured segments at the upper body. The foot sensors provided very weak correlations (0.20–0.27). Accelerations measured at the pelvis during jumping exercises can be tracked very well on the upper body and upper extremities, including locations where smart devices are typically worn, which gives possibilities for remote and continuous monitoring of programs.

## 1. Introduction

The prevalence of osteoporosis has increased significantly in recent years. Worldwide, about 33% of women and 20% of men over age 50 will experience osteoporosis fractures [1,2]. The first projections for the year 2040 indicate a doubling of acute cases [3,4]. Exercise is a key preventive strategy recommended to reduce the risk of osteoporosis, falls, and fractures [5]. However, while the preventive necessity of physical activity increases with age, the actual physical activity decreases [6,7]. Lockdowns and other restrictions, which could be seen during the Corona pandemic, also promote physical inactivity, even among younger people [8,9]. Additionally, healthcare services, such as doctor’s appointments or physical therapy, were not allowed or skipped [10]. To ensure lasting success, timely and comprehensive follow-up of osteoporosis patients is necessary to monitor treatment success or potential problems and to offer individualized rehabilitation programs to the patient [11,12]. Pandemic-induced social regulations revealed the need for more independent ways of diagnosis and especially disease prevention. However, regular outpatient and medical visits are very time-consuming and costly. Smart wearable devices offer a possible solution by combining more and more features to monitor daily movements and interactions [13,14,15], giving patients a chance to follow their progress on demand. Inertial measurement units (IMUs) are a popular kind of sensor to use in such smart wearable devices. IMUs enable the recording of body movements and body part accelerations on which the sensor is worn.

High-impact exercises (e.g., running, jumping, drop jumps) have been shown to affect bone mineral density (BMD), especially of the femur in premenopausal (age 35–40 years) women [16,17]. To verify this, all daily accelerations of the body were recorded using a body monitor (measurement based on IMU) placed at the hip for 12 months. Results showed a positive correlation between a minimum number of movements with specific high accelerations and an increased BMD in examined bones which indicates a preventive effect on osteoporosis [18,19,20]. Another study shows positive effects of a nine-month jumping intervention on BMD and content in non-osteogenic sports, such as swimming and cycling [21].

A positive effect between jumping exercises and osteoporosis has been demonstrated, with the underlying kinematics and possibilities to track those in a natural setting still being interesting topics. Thus, our conducted study combines two parts to further investigate this topic—a rather mechanical/theoretical part to validate the effectiveness of different body parts to measure and identify those jumping exercises and secondly, a more in-depth analysis of the kinematic and preventive aspects focusing on osteoporosis. The presented manuscript focuses only on the first topic.

Single acceleration data or the combination of acceleration data measured at different points allow drawing conclusions about certain movements and their effects on the body. This study aims to evaluate acceleration curves of different body segments during high-impact exercises (jumps) to understand the kinematics behind the positive effects of jumping exercises on osteoporosis.

The intention is further to clarify at which segments or body regions jumping-exercise-based impacts on the lower extremities can best be recorded compared to findings on hip worn measurements. Can these loads be tracked at the locations where smart devices are typically worn, such as the wrist (smartwatch), upper leg (smartphone), or sternum (chest strap), which are more pleasant and more manageable in everyday life?

## 2. Materials and Methods

### 2.1. Participants

Forty-nine participants were recruited for this study. All of them granted informed consent. BMD peaks at around age 30. Women lose BMD faster than men. After menopause, the risk of developing osteoporosis increases in women. The age ranges were chosen to include subjects in whom BMD tends to begin to decline, but women have not yet reached menopause. The upper age limit was standardized for all genders [22,23]. Therefore, the criteria for taking part in this study were set to an age of 30 to 45 years, two hours of physical activity per week, and no musculoskeletal condition, and injury for the last 12 months. The idea was to limit the risk of injuries during different jumping tasks. The main kind of sports the subjects usually did were running, cycling, and weight training (multiple answers were possible). Data were collected over two weeks, one week at the Laboratory for Biomechanics at the OTH Regensburg and one week at the Greifswald University Medicine. A list of subject characteristics is given in Table 1.

### 2.2. Experimental Setup

The jumping exercises were recorded at 60 Hz using 17 IMUs (MVN Awinda, Xsens Technologies B.V., NL) for a full-body setup, comprising a 3-axial accelerometer, gyroscope, and magnetometer. The sensors were placed on the head, shoulders, upper sternum, pelvis, upper and lower legs, and feet with Velcro straps. Besides the motion capture data generated via sensor fusion algorithms, specific parameters, like acceleration or orientation, can be analyzed separately to imitate smart devices worn on the body. A 30 cm-high wooden box served as a raise to perform the drop jumps. The height was chosen to give the subjects a safe feeling when performing the exercise. Due to the different physical activities performed in their free time, some subjects were less familiar with jumping exercises. The aim was to keep the risk of injury low and still maintain a high impact on the bones. Previous studies suggest not going higher than 30 cm [24,25].

**Figure 1:**
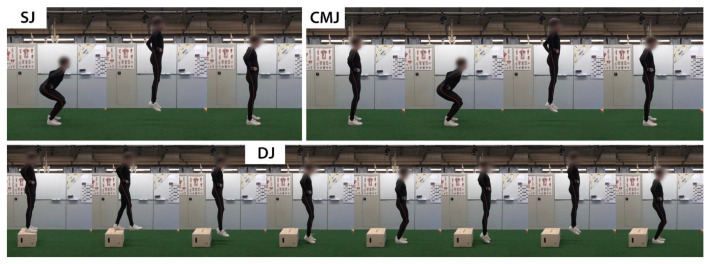
Step-by-step execution of the different high-impact exercises SJ, CMJ, and DJ. The hands must remain on the hips during the entire execution. When landing, ensure as little cushioning as possible through knee flexion. The height of the box at the DJ is 30 cm.

The procedure of the experiment was explained to the participants, and their consent was obtained before recording. Demographic and anthropometric data of the subjects, such as age, gender, weight, height, were collected, as well as physically active time and previous musculoskeletal injuries in the last two years. The subjects performed different jumping tasks five times each, containing Squad Jumps (SJ), Countermovement Jumps (CMJ), and Drop Jumps (DJ). The exact execution of the exercises is shown in Figure 1. Attention should be paid to not cushioning the landing by strong knee flexion for all exercises but to keep the legs as extended as possible in order to achieve as high as possible the impact on the bones [26]. The first landing/ground contact when executing the DJ is followed by a bounce hop. The second landing follows the instruction of the SJ and CMJ. In contrast to the SJ and CMJ, the jump height for the DJ is mainly achieved by reflex plantar flexion of the ankle joint with the knee angle as extended as possible.

Before every exercise, the right execution was demonstrated, and the subjects were allowed to do a test run first to get used to the task and the system. The subjects had to keep their hands on their hips during the exercises. If the execution was not done right, the subjects were asked to repeat the trial.

### 2.3. Data Processing

Motion capture data from Xsens were reprocessed by MVN Analyze software (version 2021.2; Xsens, Enschede, The Netherlands) (MVNA). Further data processing and analysis were performed using Python (Python Software Foundation; Python Language Reference; version 3.8.3) with the packages numpy (v. 1.18.5), pandas (v. 1.0.5), seaborn (v. 0.10.1), and scipy (v. 1.7.2). Acceleration values in gravitational direction for all 17 IMUs, were extracted from the MVNA post-processed files for all subjects (49), exercises (3), and trials (5). Only accelerations in gravitational direction were considered, as the impacts of the jumping exercises affect the body in the gravitational direction. The execution of the jumps takes place without significant movements in the transverse plane. In the first step, the measurement data were cut down to the period of the actual exercise. Subsequently, trajectories of the acceleration graphs between the different sensors and the three jumping exercises were compared according to the highest accelerations, as these maxima can be equated with a high impact on the body or bone. The maximum jump height for each trial was evaluated using MVNA software.

In the further course, the jumping exercises were considered and evaluated separately. The Pearson correlation coefficients ρ were calculated for each possible combination of the time series of the pelvis sensor and the 16 other sensors for each subject and trial. As correlation coefficients are not unbiased, the average of several correlations will not converge to the true correlation. Therefore, the correlation coefficients are transformed with the Fisher z-transformation, then averaged, and finally, calculated back into the Pearson correlation coefficient. The Pearson correlation coefficients were interpreted according to Cohen (1988), where ρ ≤ 0.29 should be considered as low correlation, 0.30 ≤ ρ ≤ 0.49 as moderate correlation, and 0.50 ≤ ρ as strong correlation [27].

## 3. Results

**Figure 2:**
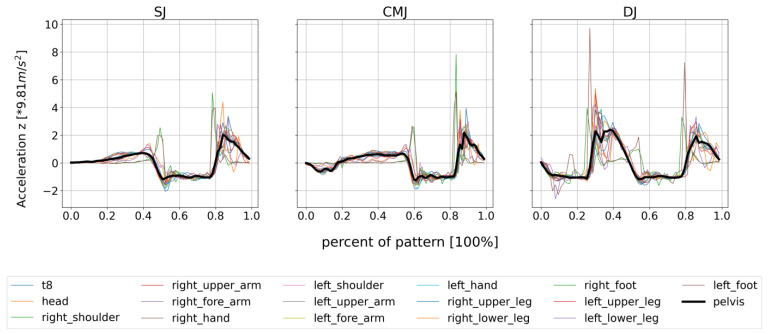
Acceleration curves of all 17 sensors during the jumping exercises, illustrated on a single subject for the third trial each. The acceleration is given as a multiple of the acceleration due to gravity.

The graphs of the acceleration in gravitational direction show very similar curves for all sensors. Positive accelerations in the graphs spatially mean a movement upwards (jump) or the deceleration of the movement at the landing. Clear outliers can only be observed for the accelerations of the foot sensors. The peak accelerations here are partly two times that of the remaining sensors. For the DJ, however, the differences are smaller. In order to illustrate what has been described, the graphs of the accelerations for one randomly selected person are shown in Figure 2.

**Figure 3:**
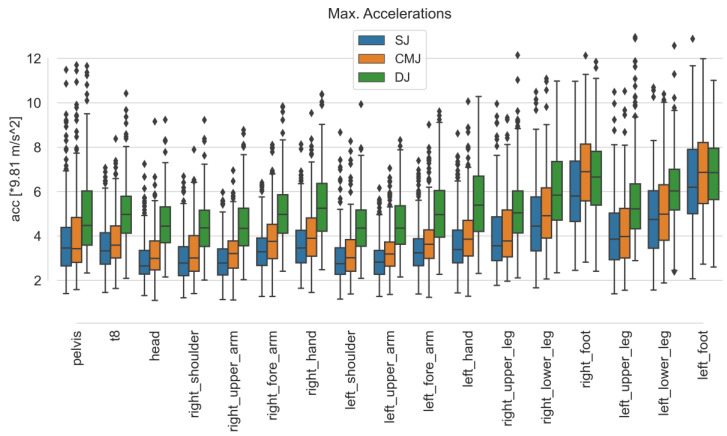
Boxplots of maximum accelerations in gravitational direction over all subjects and trials for each sensor and exercise.

Comparing the maximum accelerations of the sensors for each exercise, the medians for DJ are higher than for SJ and CMJ. The second-highest accelerations occurred for CMJ. The comparison of the sensors for the exercises is shown in Figure 3. For the sensor on the left forearm (imitating a smartwatch), a median of 3.23 for SJ, 3.62 for CMJ, and 4.9 times the acceleration due to gravity for DJ was obtained.

The evaluation of the drop height shows, on average, 0.37 ± 0.06 m (min: 0.23 m, max: 0.54 m) for the SJ and 0.41 ± 0.07 m (min: 0.3 m, max: 0.59 m) for the CMJ. For these two jumps, the maximum jump height is evaluated, which results in the drop height. This is achieved by the subject’s jumping power. For the DJ the average maximum drop height of 0.47 ± 0.02 m (min: 0.42 m, max: 0.55 m) corresponds to the height after jumping off the 30 cm high box and not after the bouncing hop.

**Figure 4:**
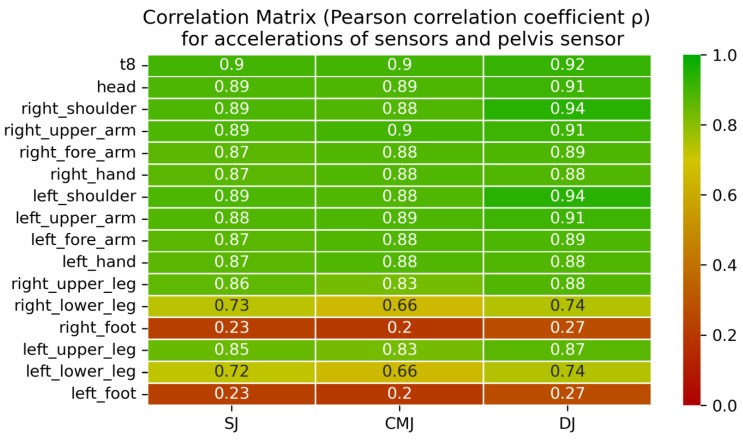
Correlation matrix for the acceleration of the pelvis sensor and one other sensor in gravitational direction for SJ, CMJ, and DJ. The values correspond to the correlation of the respective sensor combinations concerning the acceleration curves for each exercise. The color scale underlines the strength of the correlation.

Pearson’s correlation matrix over the whole time series shows that the measured accelerations in the direction of gravity correlate very well for all exercises between the pelvis sensor and sensors at the upper body. Here, all correlation coefficients are above 0.87 (min: 0.87, max: 0.94). For the thighs, the correlation with the pelvis sensor is still above 0.83 (min: 0.83, max: 0.88). Accelerations in the lower leg correlate less well with the pelvis. The coefficients here take values between 0.66 and 0.74. The foot sensors do not correlate with the upper body with values below 0.27 (min: 0.20, max: 0.27). A heat map pointing out the correlation strengths is shown in Figure 4.

## 4. Discussion

In this study, acceleration data from 17 IMUs recorded during three different jumping exercises were analyzed and evaluated. The results show that the maximum accelerations experienced by the body or the individual body segments depend on the jumping exercise performed. The DJ exerts the highest accelerations on the body. The lowest impacts are achieved by the SJ. This gradation can be attributed to the different drop heights, which are the highest for the DJ. Another reason for the increased accelerations over the entire body could be the bounce hop, in which the impact is mainly absorbed by the ankle joint. The knee does not flex as much here as it does in the other two exercises. The high accelerations in the foot sensors can be explained by the kinematic chain. The feet are the first segments of the body to be decelerated when landing. All other segments are cushioned distally via the intermediate joints (ankle, knee, hip) and the muscles running above them. They are also very unstable until they finally come to rest flat on the ground. The strong deviations in the curve to the other sensors can be attributed to this.

These deviations can also be seen in the correlation coefficient matrices shown in Figure 3. Sensor correlation combinations including a foot sensor have the lowest correlations for all exercises. However, a very good correlation can be read for all combinations between the pelvis and sternum, shoulders, upper arms, forearms, and hands, starting at values of 0.87. To reach these correlation coefficients, the hands must remain on the hips throughout the exercise. None of the previously mentioned segments of the upper body and their connecting joint angles are actively involved in cushioning the movement. Therefore, they all experience very similar accelerations on landing. Slight differences can be explained by the damping effect of the vertebral bodies or compensating movements to maintain balance.

The maximum accelerations at the different sensor positions indicate that the impact of the jumping exercises can be measured with an acceptable accuracy on almost all parts of the body. When these results are compared with previous studies, the mean values tend to be lower in the present study. This can be attributed to the lower sampling rate, which has the disadvantage that very short acceleration peaks may not be detected. Vainionpää et al. (2006) used an IMU with a sampling rate of 400 Hz [19]. The acceleration data in this study was acquired at a frequency of 60 Hz. This sampling rate was chosen to compare with typical values of wearables [28,29,30]. The maximum accelerations are still in the same areas, thus, these peaks were sufficiently captured.

This study compares different jumping exercises whose execution is predetermined. Therefore, the results cannot be generalized. Other movements or movement patterns must be evaluated separately, as the extremities experience different accelerations during other movements. In addition, the Xsens sensors cannot be accurately compared to smart devices, even if the placement can be assumed to be the same. In this study, the sensors were fixed to the skin with Velcro straps. A consistent position of the sensor is important, as can be assumed with a smartwatch, for example. Smartphones, on the other hand, are not so well suited, as they have degrees of freedom in the trouser pocket, which can lead to wrong results. Specifications of wearables must be observed before use. They must be able to record the same parameters, and these parameters should also be retrievable to evaluate the performed exercises.

The characteristics of the test persons were very different in this study. Especially the different physical conditions and training levels should be emphasized, which made a controlled execution of the exercises difficult due to a lack of coordination and jumping power. Having this wide range of subjects reinforces the high correlations. Nevertheless, it leaves room to examine the results in detail under the aspects of age, gender, and weight in order to be able to make even more specific statements.

## 5. Conclusions

The study shows that the accelerations measured at the pelvis during jumping exercises can be tracked very well on the upper body and upper extremities. It sets a basis regarding the kinematic relationships of different body segments during these jumping exercises. This includes the locations where smart wearable devices are typically worn, such as the wrist (smartwatch) or sternum (chest strap). Sensors positioned at the thighs do not offer as good correlations as the upper body and extremities but are still strong. If attention is paid to the correct execution of the exercise, the sensor placement is almost arbitrarily variable. These results suggest that these jumping exercises can be used in possible wearable app combinations to assist with the execution and provide important information regarding impact. As shown in past studies, these jumping exercises are sufficient to have a positive effect on BMD. The impact forces and the exact preventive kinetic mechanisms regarding osteoporosis are difficult to deduce from the accelerations measured at the upper body. Therefore, possible further kinematic and kinetic correlations are to be clarified in future investigations.

## Figures and Tables

**Table 1:** Subject demographic and anthropometric characteristics. Most performed sports were queried (multiple responses were possible).

Variable	Value
Sex	
Female	22
Male	27
Age (years)	33.7 ± 4.2
Height (cm)	174.0 ± 7.5
Weight (kg)	74.1 ± 13.4
BMI (kg/m^2^) Sports	24.3 ± 3.3
Running	25
Cycling	18
Weight training	11
Dominant Leg	
Left	3
Right	46

## Data Availability

The data analyzed and presented in this study are available upon reasonable request from the corresponding author.
